# Efficacy of erbium-doped yttrium aluminium garnet for achieving pre-emptive dental laser analgesia in children: A study protocol for a randomized clinical trial

**DOI:** 10.1097/MD.0000000000013601

**Published:** 2018-12-21

**Authors:** Elitsa Veneva, Ralitsa Raycheva, Ani Belcheva

**Affiliations:** a Department of Pediatric Dentistry, Faculty of Dental Medicine; b Department of Social Medicine and Public Health, Faculty of Public Health, Medical University – Plovdiv, Bulgaria.

**Keywords:** erbium-doped yttrium aluminium garnet anesthesia, laser analgesia, low-level laser therapy, photobiomodulation, photomodulation

## Abstract

**Introduction:**

A current non-pharmacological mean for attaining painless conservative treatment is presented by laser analgesia (LA), considered as bio-photomodulation of pulp reactivity aiming reduction of nociceptive impulse formation. Currently no consensus is reached regarding a detailed protocol with laser parameter settings for pre-emptive LA. The purpose of this study is determining the efficacy of erbium-doped yttrium aluminium garnet (Er:YAG) laser in achieving pulpal analgesia and quantifying duration and extent of any effects assessed.

**Methods and analysis:**

The proposed study is a double-blind placebo-controlled randomized split-mouth clinical trial with 2-way repeated measures design. Eligible patients of age 10 to 12 years undergo 2 single-visit treatments, receiving LA or placebo analgesia (PA) prior to caries ablation, randomized via computer-generated, permuted-block sequence. Primary outcome measure is pain felt during treatment, reported by patient on visual-analogue scale. Secondary outcomes: changes in pulpal sensibility to electrical and cold-stimuli; patient experience during LA/PA; pain-related behavior according to Faces, Legs, Activity, Cry, Consolabilty (FLACC) scale; heart-rate dynamics. Data will be analyzed with intention-to-treat concept by Student *t* test for paired samples, *P* < .05. Pre-test on 20 subjects resulted in n = 41 patients needing to be recruited.

**Ethics and dissemination:**

This study protocol has been approved by the Committee for Scientific Research Ethics, Medical University - Plovdiv, Bulgaria (Reference number P-8604, Protocol of approval N:6/23.11.2017) and registered on a publically accessible database. This research received institutional funding from the Medical University – Plovdiv, Bulgaria under project SPD-03/2017. Findings will be reported in scientific publications and at research conferences, and in project summary papers for participants.

**Trial registration:**

ClinicalTrials.gov (Registration number: NCT03412721).

## Abstract 2

Supplemental Digital Content is available in the text

## 1 Introduction

### 1.1 Background and rationale

Achieving local anesthesia in children is one of the critical aspects of pain management. A current non-pharmacological mean for attaining painless conservative treatment is presented by the laser analgesia (LA) method.

LA is a non-invasive, non-thermogenic bio-modulation of the dental pulp reactivity aiming for reduction of impulse formation of the pulpal nociceptors. It is hypothesized that laser pulses alter the cell membrane behavior of the pulpal nerve fibers by hyperpolarization and loss of impulse conduction, and thus an analgesic effect is achieved.^[1–3]^

The assessment of changes in pulp's sensory responses can be explored by pulp sensibility testing,^[4]^ such as one delivered through thermal or electric test. The aforementioned tests could be valuable means for investigating the occurrence of any pulpal analgesic effect obtained by dental lasers.

The A-delta nociceptive nerve fibers in the pulp are able to generate a fast, sharp pain that is easily localized^[5,6]^ such as one from rapid temperature change,^[7]^ achieved by application of Cold-test. Еlectric pulp testing (EPT) depends on ionic movement and triggers the A-delta fibers as well, due to their conduction speed and their myelin sheath.

The rationale based on the neurophysiology of the pulp, led to choosing both electric and cold testing as means for evaluating the pulpal analgesic effect of the erbium-doped yttrium aluminium garnet (Er:YAG) pulp laser. To our best knowledge, no study before has implemented the use of Cold-test to complement EPT-results in assessment of LA efficacy.

The pulp sensibility testing, along with the assessment of subjective and objective pain sensation during treatment should help estimate the clinical adequacy of the LA method by finding out if pain-free operative treatment can subsequently be performed.

### 1.2 Objectives

The intention of the technique of “pre-emptive LA” is to reduce sensation in that small percentage of patients who may experience unpleasant sensations during caries removal. We hypothesized that when operating at low level densities, the laser energy leads to loss of nociceptive impulse formation by coinciding with the natural resonance frequency (15–20 Hz)^[8]^ of cell membranes of nerve fibers in the dental pulp, leading to an analgesic effect.

The aim of this study is efficacy approbation of a modified protocol for LA with Er:YAG for achieving pulpal analgesia in pediatric patients and quantification of the duration and extent of any effects assessed.

The main objectives are to compare pain felt during treatment in laser and placebo analgesia (PA) control group and to register the reactivity of the pulp towards cold and electrical stimuli before and after inducing laser or PA. The second objectives are to evaluate latency of any analgesic effect, patient experience during analgesic or placebo procedure, as well as heart rate dynamics and need for additional anesthesia during treatment.

### 1.3 Trial design

**Figure 1:**
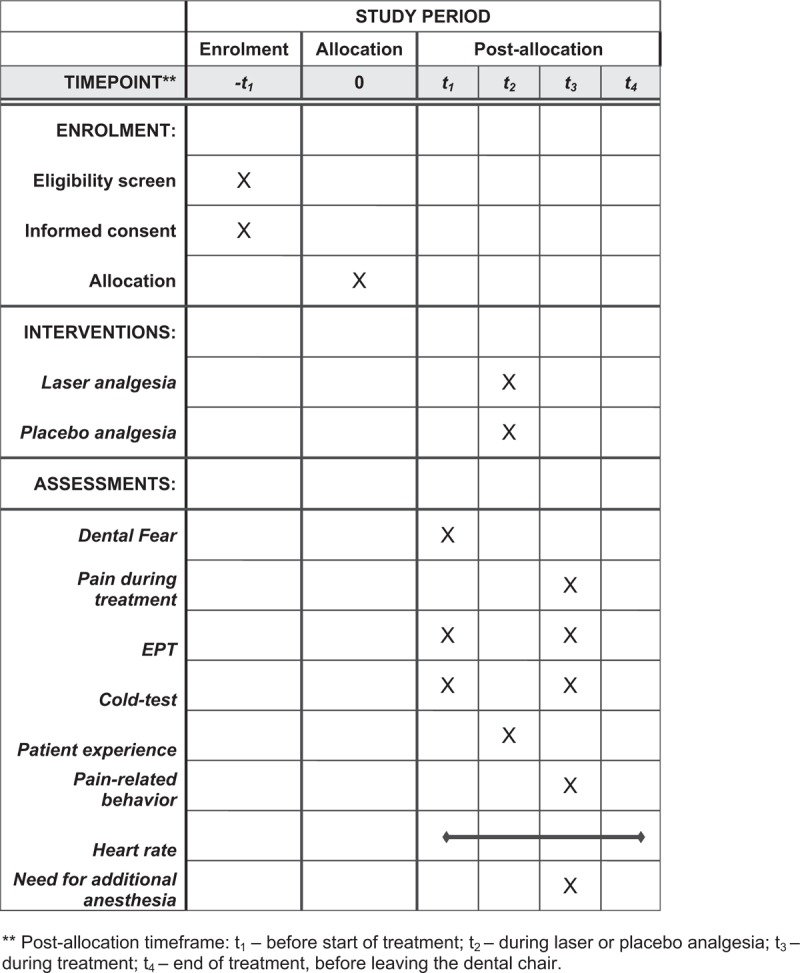
Schedule of enrollment, interventions, and assessments of treatments.

The trial to be conducted is a double-blind controlled clinical crossover experimental study with 2-way repeated measures design. Figure 1 summarizes the enrollment, intervention, and assessment schedule, all of which are in accordance with the Standard Protocol Items for Randomized Trials (SPIRIT) recommendations,^[9]^ shown in Table 1 . Patients and outcomes assessor are blinded for the study. Experimental group in this study consists of permanent upper jaw first molars, receiving pre-emptive LA prior to laser caries ablation, whereas control group consists of contralateral permanent upper jaw first molars with similar defects of the same patient, receiving placebo-analgesia prior to laser treatment.

**Table 1:**
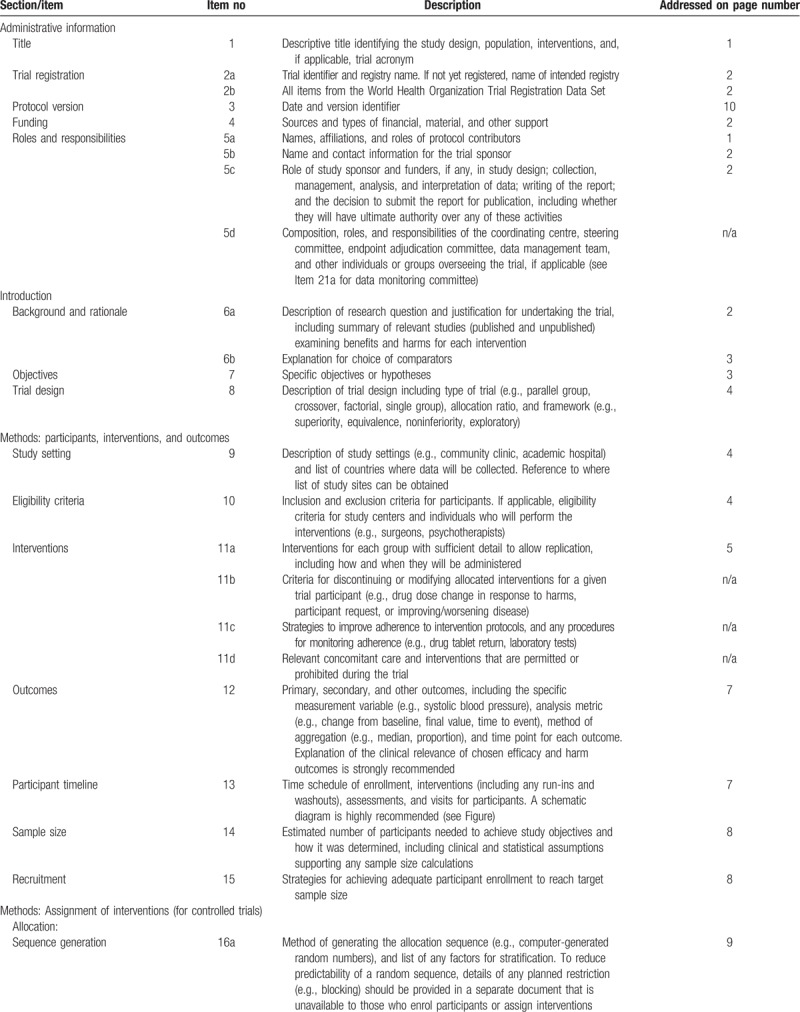
SPIRIT 2013 checklist: recommended items to address in a clinical trial protocol and related documents^∗^.

**Table 1 (Continued):**
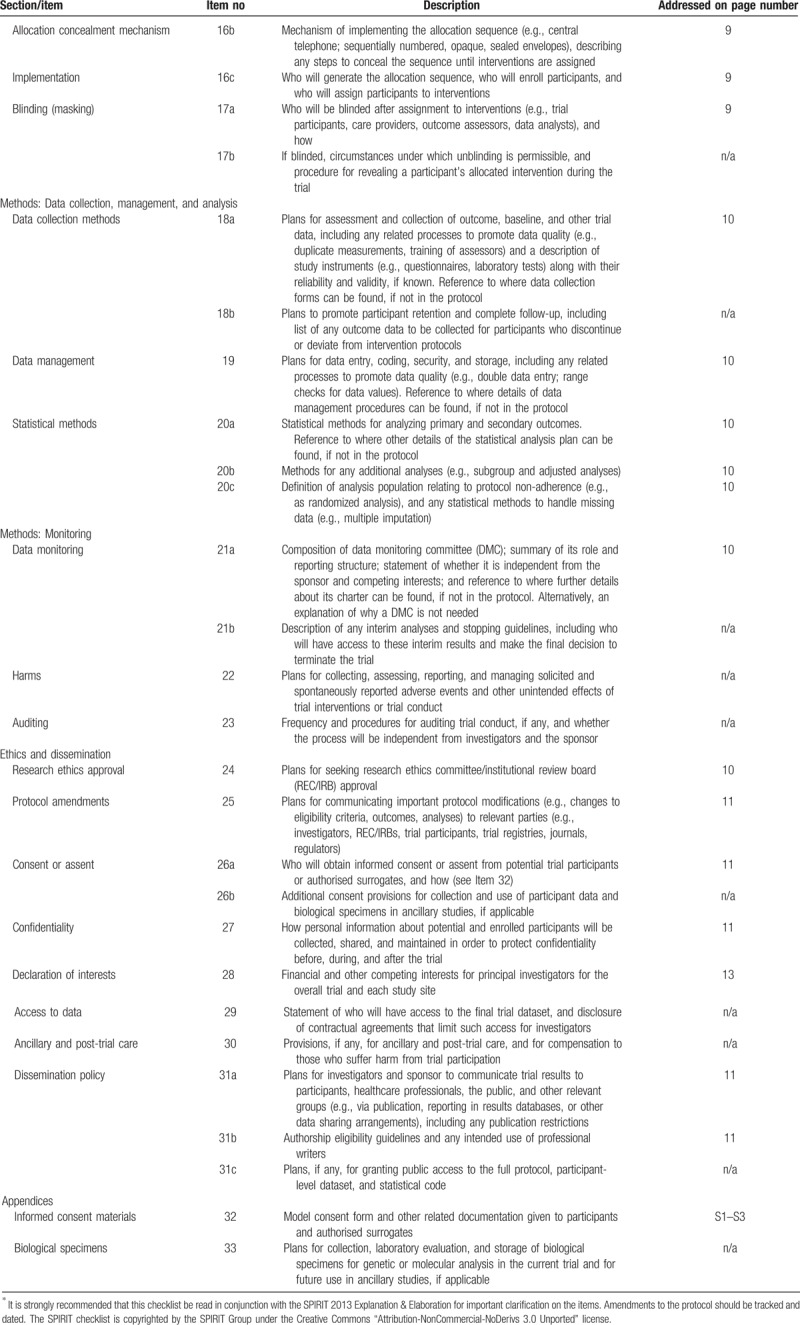
SPIRIT 2013 checklist: recommended items to address in a clinical trial protocol and related documents^∗^.

The required number of patients is calculated on the basis of a micro-sample at an accepted level of significance (*P* < .05) and a maximum permissible error *α* < 0.05 and *β* < 0.2. Intention-to-treat concept is chosen as statistical approach for data analysis.

## 2 Methods and analyses

### 2.1 Study setting

The setting of this trial is the Department of Pediatric Dentistry at the Faculty of Dental Medicine, Medical University of Plovdiv, Bulgaria.

### 2.2 Eligibility

#### 2.2.1 Inclusion criteria

1. Participants in the study are children 10 to 12 years old, compliant with the cognitive development of the child and the requirement for full root development for diagnostics with electrical pulp testing.
2. Children, identified as positive or definitely positive through Frankl behavioral rating scale.
3. Children who are not considered medically compromised or medically complex patients. The absence of disease is confirmed by anamnestic interview with a parent or a care-giver of the child and excludes general acute or chronic disease, cognitive impairment.
4. Patients, requiring conservative treatment of occlusal or foramen caecum caries on 2 first permanent upper jaw molars without prior restorations or dental sealants. Lesions are to be classified as moderate caries by the International caries detection and assessment system (ICDAS) with code 03 or 04, which do not present spontaneous unprovoked pain, percussion or palpation pain or other symptoms, indicating of pulpal or periodontal pathology. Included are carious lesions only on vital teeth, involving up to half of the dentine thickness.
5. Obtained informed consent from parents or gave-givers to participate in the study, in which procedures are explained in appropriate manner (see supplementary data file S1—“Informed consent” and supplementary data file S2—“Information leaflet”).

#### 2.2.2 Exclusion criteria

1. Patients who are undergoing therapy with neurological, sedative, analgesic, and/or anti-inflammatory drugs 7 days prior to treatment.
2. Children, who are first time ever dental patients.
3. Patients who are undergoing treatment or have been treated 6 months prior to inclusion with remineralizing agents.
4. Excluded are first molars which are affected by hypoplasia or hypomineralization.

#### 2.2.3 Interventions

Dental Er:YAG laser (LiteTouch, Light Instruments LTD) will be used as means to attain analgesia and caries removal. Chosen protocol parameters are modified based on previously conducted studies.^[8,10,11]^

Laser analgesic protocol: Water mist spray set to “maximum,” non-contact handpiece with sapphire tip. Tip-to-tissue distance 10 mm from the tooth neck, achieved by using a spacer. Energy is delivered to the enamel above the gingival margin adjacent to the cemento-enamel junction (perpendicularly towards the dental pulp) on each of the 4 line angles of the tooth for 30 seconds, moving the laser handpiece in a sweeping action. Pulse energy—0.2 W/10 Hz/20 mJ. Follows increase of energy and repetition of protocol—0.6 W/15 Hz/40 mJ. Total duration of LA-induction—240 seconds.

Placebo analgesic protocol: No pulse energy applied. Moving the laser handpiece in a sweeping motion repeating actions to imitate LA placement.

Application parameters during caries ablation: Hard tissue preconditioning: 1.5 W/15 Hz/100 mJ for 1 minute; Enamel removal—3 W/15 Hz/200 mJ; Dentin removal—2 W/10 Hz/200 mJ. Smear layer removal: 2 W/10 Hz/200 mJ. Loss of tooth structure was restored with esthetic composite.

#### 2.2.4 Clinical protocol:

First visit:

1. Parents or care-givers are informed about laser technique and get acquainted with the nature of the research being conducted in to prepare their children for the dental treatment. The parent or care-giver signs informed consent.
2. Patients are asked to complete Children's Fear Survey Schedule–Dental Subscale (CFSS-DS) questionnaire.
3. Pulse-oximeter is connected to patient's index finger. Start of heart rate monitoring and recording—7 minutes prior treatment. Time frame: until end of treatment.
4. Blind for chosen method investigator evaluates the initial reactivity of the pulp with EPT 5 minutes prior laser or PA. Four minutes before applying chosen method same investigator performs Cold-test with propane-butane gas, applied on a cotton pad on the tooth. Patient is asked to evaluate pain perception on VAS.
5. The chosen method, placebo or LA, is applied.
6. Patient experience during LA/PA is evaluated by a patient questionnaire immediately after the procedure. Patient is asked to answer 4 questions with possible answers “yes” and “no”: “Did you feel pain when we put your tooth to sleep?”; “Were you frightened when we put your tooth to sleep?”; “Do you feel any pain now that your tooth is put to sleep?”; “Do you feel any tingling sensation now that your tooth is put to sleep?”.
7. Laser ablation of the carious lesion begins. No rotary instruments or other excavation techniques are to be applied.
8. Five minutes after the analgesic procedure same investigator evaluates the sensibility of the pulp with EPT and 6 minutes after performs Cold-test on same tooth. Patient is asked to evaluate pain perception after Cold-test on the VAS pain scale.
9. At the 20th minute after LA/PA, EPT testing is performed. At the 21st minute a Cold-test is applied and the pain perception is registered on the VAS.
10. Outcomes assessor monitors the patient during treatment and registers pain related behavior using Faces, Legs, Activity, Cry, Consolability (FLACC) Behavioral Pain Rating Scale.^[12]^
11. Patients who complete the procedure without the need for additional anesthesia, immediately after placement of restoration are asked to use the combined VAS scale to quantify the level of pain felt during treatment. Patients who request that the procedure is terminated to administer an anesthetic injection, are asked to rate their level of pain on VAS, immediately following termination.

Second Visit:

Application of PA/LA prior to treatment of adjacent first molar of upper jaw, according to treatment allocation sequence number. Reactivity of the pulp to cold and electric stimulation, as well as the subjective sensation after manipulation by the aforementioned methods are reported.

### 2.3 Outcomes

#### 2.3.1 Primary outcome measures

Primary outcome measure to be assessed in this study is pain felt during the treatment, reported by the patient on a visual analogue scale (VAS) at the end of the treatment session.

#### 2.3.2 Secondary outcome measures

Assessment of following secondary outcomes will be performed: changes in pulpal sensibility to electrical stimuli before and after LA/PA, evaluated by EPT (time frame: 25 minutes); changes in pulpal sensibility to cold stimulation before and after laser/PA by Cold-test—self-reported pain by the patient on VAS (Time Frame: 25 minutes); patient experience during LA/PA, evaluated by questionnaire; pain-related behavior during treatment, evaluated using the FLACC scale; heart-rate dynamics during the experiment, registered via pulse oximeter; need for additional local anesthesia infiltration.

#### 2.3.3 Participant timeline

Each eligible patient undergoes 2 single-visit treatments of the 2 carious first upper jaw molars, receiving LA when undergoing treatment of 1 tooth and PA at the other visit during treatment of the homologous contralateral tooth. The 2 manipulations are performed by 1 operator, performing treatment of caries and restorative procedure. Outcomes are registered by the primary investigator in a clinical file (see supplementary data file S3 - “Clinical file”). A 7 to 21 day interval is allowed between one procedure and the other. The protocol to be used in the first procedure is randomly selected using a computer-generated list linked to sequence of enrollment in the trial.

#### 2.3.4 Sample size calculation

Given the lack of comparable research and the unknown population standard deviation we conducted a pretest with 20 subjects and considered the behavior of this subgroup as population estimate. To estimate sample size for the primary outcome—pain felt during laser ablation of moderate carious lesion, according to the VAS scale—we applied a *t* test for paired groups (G∗ Power software version 3.1),^[13]^ since we have 2 groups (first upper molar in right and left quadrant) on the same patient.

The effect size was determined using the formula 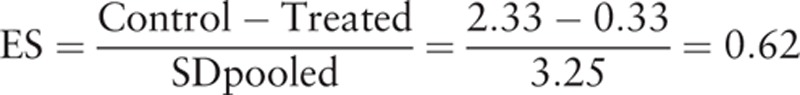


Where SD is the pooled standard deviation—an average of the standard deviations of the experimental and control groups. The error was set at 5% and the power test at 95%. According to the calculation, a sample of 37 patients will be necessary to detect differences in pain. Since drop-outs are unavoidable when collecting follow-up data, this number needs to be adjusted for the estimated drop-out rate. During the pre-test, we had a drop-out rate of 5% for collecting the follow-up data and if we anticipated a higher drop-out rate for this study, we conservatively allowed for a 10% drop-out rate. Adjusting the sample size for this drop-out rate results in a sample of 41 patients needing to be recruited.

### 2.4 Recruitment

The clinical trial is currently recruiting participants. Patient recruitment started in October 2018. Estimated study completion date is May 1, 2019. The enrollment capacity was estimated to be 6 patients/mo.

### 2.5 Participating centers

Eligible patients are selected from the visitors of the pediatric dental clinic of the Department of Pediatric Dentistry, Faculty of Dental Medicine—Medical University of Plovdiv, Bulgaria and treated in the laser dental office of the aforementioned.

### 2.6 Assignment of interventions

#### 2.6.1 Sequence generation

A computer-generated, permuted-block randomization sequence for allocation of first procedure is to be prepared. Patients are to be randomized to treatment allocation according to number of enrollment in the trial. In this split-mouth randomized controlled trial (RCT), every patient will receive both procedures. The patient will be randomized to receive at the first visit, placebo analgesic procedure and, at the second visit, laser pre-emptive analgesia or at the first visit—LA and at second one—placebo procedure.

#### 2.6.2 Allocation concealment mechanism and implementation

Randomization is based on treatment allocation sequence number. First procedure (PA—1; LA—2) is linked to number of enrollment in the study in Microsoft Excel table. Allocation sequence will be generated before start of the patient enrollment by the statistician. The operator will obtain each randomization allocation via a sequentially-numbered opaque sealed envelope prior to treatment, enabling the sequence to be concealed until the intervention is assigned. The outcomes assessor will not be aware of chosen treatment. Patients will be enrolled by the primary investigator.

#### 2.6.3 Blinding

The current study is designed as a double-blind trial. Patients as well as outcomes assessor are blinded for the study. The operator will get acquainted with procedure to be performed prior to treatment session. The clinicians involved in this study as operator and outcomes assessor are selected to be the only ones performing the manipulations in order to prevent bias.

#### 2.6.4 Data collection, confidentiality, storage, and monitoring of study documents

According to the regulations of the Personal Data Protection Act, the collected paper forms will be stored in a secure manner in the Department of Pediatric Dentistry, Faculty of Dental Medicine, Medical University - Plovdiv, Bulgaria. Outcomes will be transferred electronically by the primary investigator after transmission of paper forms. Clinical research files will be stored in a locked, secure office. Data will be electronically stored on a double password-protected computer. Only the primary investigator and the statistician will have access to the final data set. The trial will be monitored by the research monitoring officer of Medical University—Plovdiv, verifying that the study is conducted in accordance with the Good Clinical Practice guidelines.

## 3 Statistical methods

The unit of analysis will be the tooth for the split-mouth RCT (2 first permanent molars belonging to the same dental arch treated per patient). The data will be recorded and analyzed using SPSS 20.0. All data will be analyzed using an intention-to-treat analysis.^[14]^ Descriptive statistics will be calculated. Discrete variables will be summarized by frequencies or proportions. Continuous variables will be reported as means and standard errors or medians and range (depending on the distribution of the variables). Data will be checked for baseline differences between the treatment arms. If baseline differences do occur for any of the variables, they will be added to subsequent models to compensate for those differences using an analysis of covariance approach.

We will compare pain mean scores according to the visual analogue scale (VAS), containing numerical symbols. We will report the mean differences between groups and the associated 95% CIs. For the split-mouth RCT, with each patient being his or her own control, our statistical analysis will take into account the paired nature of data and the results will be analyzed by Student *t* test for paired samples.

## 4 Ethics and dissemination

The clinical trial will be carried out in line with the principles of the Declaration of Helsinki and according to the Clinical Trials Directive 2001/20/EC of the European Parliament on the approximation of the laws, regulations, and administrative provisions of the Member States relating to the implementation of Good Clinical Practices in the conduct of clinical trials on medicinal products for human use.

### 4.1 Research ethics approval

This study has been approved by the Committee for Scientific Research Ethics, Medical University—Plovdiv, Bulgaria (Reference number P-8604, Protocol amendment number: 01, Protocol of approval N:6/23.11.2017) and registered on a publically accessible database ClinicalTrials.gov (Registration number: NCT03412721). The protocol of the study, as well as written information leaflets and informed consent documents are approved by the Ethics committee. Should there be any changes in the aforementioned, they will be consulted with the committee.

### 4.2 Consent

Parents or caregivers will be given written informed consent and information leaflets by the primary investigator in person.

### 4.3 Confidentiality

People with direct access to the data will take all necessary precautions to maintain confidentiality. All data collected during the study will be rendered anonymous. Only initials and inclusion number will be registered.

### 4.4 Dissemination policy

The results of the study will be released to dental medicine specialists and scientific community no later than 1 year after completion of the trial, through presentation at scientific conferences and publication in peer-reviewed journals. The principal investigator (EV), the scientific expert and operator (AB), and the statistician (RR) will write the first draft of the manuscript.

## 5 Discussion

LA is a new alternative and a traumatic method that could help improving the quality of pediatric dental care. Different possible explanations have been suggested for low-level laser action (bio-photomodulation) regarding pain relief.^[15]^ Currently no consensus is reached regarding a detailed protocol with reliable laser parameter settings for pre-emptive LA, and some research lacks necessary parameter detail, presenting challenges to repeat or reproduce.^[11]^ Many studies nevertheless agree that to obtain pulpal analgesia, it is necessary to take advantage of low energy and power densities.^[3,8,11,16,17]^

The clinical adequacy of Er:YAG laser for achieving pre-emptive dental analgesia is investigated in a complex manner by pulp sensibility testing, along with assessment of subjective and objective pain during treatment of similar cases. This randomized controlled trial (RCT) trial is a well powered one-center split-mouth experimental study with two-way repeated measures design. The trial is double-blind, where patients and outcomes assessor are blinded for the study, involving only one operator, who cannot be blinded, and one primary investigator, reducing inter-individual variability from the estimates of the treatment effect. A disadvantage of this trial is the need to include positive patients with symmetrical and similar conditions, and many patients are not eligible. The following precautions are taken in account to minimize variables in results: eligible patients are selected from a narrow age group (10–12 years), classified as positive or definitely positive through Frankl behavior scale. Age of the patients is compliant with the need for full root development of teeth to be examined with EPT. Carious lesions are restricted to moderate caries diagnosed by ICDAS with code 03/04 on first upper molars without previous restorations. Focus of the trial is pain during treatment after pre-emptive laser or PA—the VAS scale is selected as means for subjective pain rating of the patient, while the FLACC scale is chosen to register pain-related behavior by the outcomes assessor.

Any alteration in the sensibility of the pulp after laser or placebo analgesic procedures is to be investigated through EPT and cold testing. The EPT is commonly used for assessing pulpal sensibility because it is quick and reproducible and does not appear to cause pulpal damage.^[16]^ Few clinical studies have investigated the method of LA by analyzing alteration in EPT threshold, but results are contradictory.^[16–19]^

It is possible that the EPT does not provide an accurate measure of pulpal analgesia^[16]^ and it may be preferable to assess the clinical adequacy of a dental analgesia by supplemental methods such as thermal stimulation. According to authors,^[20]^ ideally, EPT should be used in conjunction with cold testing so that the results from one test will verify the findings of the other test. Cold testing causes contraction of the dentinal fluid within the dentinal tubules by creating “hydrodynamic forces” acting on the A-delta nerve fibers, leading to a sharp sensation lasting for the duration of the thermal test.^[21]^ The rationale behind the chosen test supports our hypothesis that if any analgesic effect is attained, then the patient is expected to report lower cold-related pain VAS-scores. To our best knowledge, no study before has explored the analgesic effect of dental laser with Cold-test, complementing the EPT results.

The pulp sensibility testing, along with the assessment of subjective and objective pain sensation during treatment of similar cases should help estimate the clinical adequacy of the LA method by finding out if pain-free operative treatment can subsequently be performed.

### 5.1 Trial status

This trial is currently recruiting patients. Patient recruitment started in October 2018 and by November 2018 10 patients are enrolled. Pre-test on 20 subjects resulted in n = 41 patients needing to be recruited. Outcome results will be updated in clinicaltrials.gov after completion of the study, estimated due May 2019.

## Acknowledgments

The authors would like to show their gratitude to Nedelcho Sitnov and Rangel Prodanov for their conceptual and instrumental support in developing the special spacer used in this project. They are also thankful to all the parents and children for participating in the trial.

## Author contributions

**Data curation:** Ralitsa Raycheva.

**Formal analysis:** Ralitsa Raycheva.

**Investigation:** Elitsa Veneva, Ani Belcheva.

**Methodology:** Elitsa Veneva, Ani Belcheva.

**Project administration:** Elitsa Veneva.

**Writing – original draft:** Elitsa Veneva, Ralitsa Raycheva.

**Writing – review & editing:** Ani Belcheva.

Elitsa Veneva: 0000-0002-5257-4060.

Ralitsa Raycheva: 0000-0002-6417-5681.

Ani Belcheva: 0000-0002-9625-8684.

## Supplementary Material

Supplemental Digital Content
