# Supplementary material for: Efficacy of erbium-doped yttrium aluminium garnet for achieving pre-emptive dental laser analgesia in children: A study protocol for a randomized clinical trial
Source: Medicine (Baltimore). 2018 Dec 21;97(51):e13601. doi: 10.1097/MD.0000000000013601 (PMC6320132; doi:10.1097/MD.0000000000013601)
Supplement: Supplemental Digital Content [file medi-97-e13601-s001.doc]

**Informed consent**

I declare that I have read the information leaflet for the ‘Efficacy of erbium-doped yttrium aluminium garnet for achieving pre-emptive dental laser analgesia in children’ of the Department of Pediatric Dentistry - Plovdiv. I agree with my child participating in the clinical trial voluntarily. I authorize the dental practitioner to carry out the necessary examination and treatment of diseases in the oral cavity. I am informed that the treatment is completely free and that we can suspend our participation at any time without any reference to the future treatment of the child. I agree that the data and the photo material obtained from the child's review and treatment can be used only for scientific and educational purposes.

Name of parent /care-giver/

.................................................................................................,

Signature of parent /care-giver/ ................................................................................................,

Name of patient:

................................................................................................,

Address:

..................................................................................................,

Telephone number: ...................................................................

Name of the treating dentist

..................................................

Signature

..................................................

For additional information or queries regarding the trial, you can contact us on the given address, email of phone numbers.

Elitsa Veneva, DMD

Assistant Professor

Department of Pediatric Dentistry

Faculty of Dental Medicine

3 Hristo Botev Boulevard

е-mail: elitza.veneva@gmail.com

phone: +359898 424 151

Ani Beltcheva, PhD, DMD

Full Professor

Department of Pediatric Dentistry

Faculty of Dental Medicine

3 Hristo Botev Boulevard

e-mail: abeltcheva@yahoo.com

phone: +359889 528 932

**Information leaflet for the parent/care-giver**

**DEPARTMENT OF PEDIATRIC DENTISTRY**

FACULTY OF DENTAL MEDICINE – PLOVDIV

Efficacy of erbium-doped yttrium aluminium garnet for achieving pre-emptive dental laser analgesia in children

The current study is conducted in the Department of Pediatric Dentistry - Plovdiv by Dr. Elitsa Veneva, assistant professor, and Prof. Dr. Ani Belcheva, PhD - scientific coordinator.

Dear parents,

Achieving high-quality dental care in children is closely related to painless treatment and an approach that does not raise anxiety and fear.

You and your child are invited to participate in the program of the Department of Pediatric Dentistry in Plovdiv, which aims to investigate the effectiveness of the dental laser as a mean for achieving analgesia in the treatment of dental caries. Although the treatment of caries lesions with the Er:YAG laser is considered almost painless, we strive to find the way in which this action takes place.

The participation of your child in the program is voluntary. If you declare a willingness to participate, your child will be part of a group of children included in the program to investigate the possibility for painless treatment of caries with a laser.

Oral health is an important part of the child’s general health. Treatment of two affected teeth will be carried out with a dental laser in two separate visits.

To find out the laser's effects, we will record the sensitivity of the treated teeth to various stimuli, as well as your child's reactions to the treatment. Prior to treatment, he or she will be asked to fill out a questionnaire about fear of the dental environment. The manipulations will be explained to your child in age-appropriate manner using the tell-show-do technique. During the treatment, your child will be asked to show the amount of pain, if present, on a scale adapted for children.

The collected information will be kept strictly confidential and accessible only to the investigators conducting the study. The results will be presented and discussed in a scientific report on the project. When publishing in scientific literature, your name and data shall not be announced anywhere.

Thank you for your time and attention!

**CLINICAL FILE**

**Efficacy of erbium-doped yttrium aluminium garnet for achieving pre-emptive dental laser analgesia in children**

| **IDENTIFICATION NUMBER** | | | | | | | day | |  | month year | | | | | | | operator: |  |  |
| --- | --- | --- | --- | --- | --- | --- | --- | --- | --- | --- | --- | --- | --- | --- | --- | --- | --- | --- | --- |
|  |  |  |  |  |  |  |  |  |  |  |  |  |  |  |  |  | outcomes assessor: |  |  |
|  |  |  |  |  |  |  |  |  |  |  |  |  |  |  |  |  |  |  |
| **GENERAL INFORMATION** | | | | | | |  |  |  |  |  |  |  |  |  |  |  |  |  |
|  |  | Name of Patient: | | | | |  |  |  |  |  |  |  |  |  |  |  |  |  |
|  |  | First name: | | | | | Family name: | | | | | | | | | |  |  |  |
|  |  |  | | | | |  |  |  |  |  |  |  |  | | | | | |
|  |  | place of residence: (1-urban; 2-rural) | | | | | | |  |  |  |  |  |  |  |  |  |  |  |
| **VISIT:** |  | |  |  |  | **№** |  |  |  |  |  |  |  |  |  |  |  | 0 | 1 |
| **Anamnesis** |  | |  |  |  |  |  |  |  |  |  |  |  |  |  |  |  |  |  |
| **1. Age** (years) | | | | | | |  |  |  |  |  |  |  |  |  |  |  |  |  |
| **2. Gender** |  | | (1-male; 2-female) | | | |  |  |  |  |  |  |  |  |  |  |  |  |  |
|  | |  |  |  |  |  |  |  |  |  |  |  |  |  |
|  |  |  |  |  |  |  |  |  |  |  |  |  |  |  |  |  |  |  |  |


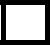


**3. Assessed behaviour of patient through Frankl Behavior Rating Scale**

1. - definitely negative
2. - negative
3. - positive
4. - definitely positive

**4. CLINICAL EXAMINATION**

5. Oral status

|  |  |  |  | 55 | 54 | 53 | 52 | 51 | 61 | 62 | 63 | 64 | 65 |  |  |  |
| --- | --- | --- | --- | --- | --- | --- | --- | --- | --- | --- | --- | --- | --- | --- | --- | --- |
| 18 | | 17 | 16 | 15 | 14 | 13 | 12 | 11 | 21 | 22 | 23 | 24 | 25 | 26 | 27 | 28 |
|  |  |  |  |  |  |  |  |  |  |  |  |  |  |  |  |  |
| ۞ |  |  |  |  |  |  |  |  |  |  |  |  |  |  |  |  |
|  |  |  |  |  |  |  |  |  |  |  |  |  |  |  |  |  |
| o |  |  |  |  |  |  |  |  |  |  |  |  |  |  |  |  |
| m |  |  |  |  |  |  |  |  |  |  |  |  |  |  |  |  |
|  |  |  |  |  |  |  |  |  |  |  |  |  |  |  |  |  |
| b |  |  |  |  |  |  |  |  |  |  |  |  |  |  |  |  |
|  |  |  |  |  |  |  |  |  |  |  |  |  |  |  |  |  |
| d |  |  |  |  |  |  |  |  |  |  |  |  |  |  |  |  |
|  |  |  |  |  |  |  |  |  |  |  |  |  |  |  |  |  |
| l |  |  |  |  |  |  |  |  |  |  |  |  |  |  |  |  |
|  |  |  |  |  |  |  |  |  |  |  |  |  |  |  |  |  |
| o |  |  |  |  |  |  |  |  |  |  |  |  |  |  |  |  |
| m |  |  |  |  |  |  |  |  |  |  |  |  |  |  |  |  |
|  |  |  |  |  |  |  |  |  |  |  |  |  |  |  |  |  |
| b |  |  |  |  |  |  |  |  |  |  |  |  |  |  |  |  |
|  |  |  |  |  |  |  |  |  |  |  |  |  |  |  |  |  |
| d |  |  |  |  |  |  |  |  |  |  |  |  |  |  |  |  |
|  |  |  |  |  |  |  |  |  |  |  |  |  |  |  |  |  |
| l |  |  |  |  |  |  |  |  |  |  |  |  |  |  |  |  |
|  |  |  |  |  |  |  |  |  |  |  |  |  |  |  |  |  |
| ۞ |  |  |  |  |  |  |  |  |  |  |  |  |  |  |  |  |
|  |  |  |  |  |  |  |  |  |  |  |  |  |  |  |  |  |

|  |  |  |  | 85 | 84 | 83 | 82 | 81 | 71 | 71 | 73 | 74 | 75 |  |  |  |
| --- | --- | --- | --- | --- | --- | --- | --- | --- | --- | --- | --- | --- | --- | --- | --- | --- |
|  | 48 | 47 | 46 | 45 | 44 | 43 | 42 | 41 | 31 | 32 | 33 | 34 | 35 | 36 | 37 | 38 |
| ***ICDAS Codes*** | |  |  |  |  |  |  |  |  |  |  |  |  |  |  |  |
| **Code restorations/sealants** | | |  | **Code caries** | | |  |  |  |  |  | **Code missing teeth** | | | |  |
| 0 | = sound |  |  | 0 | = sound |  |  |  |  |  |  | 97 | = extracted because of caries | | | |
| 1 | = sealant, partial |  |  | 1 | = first visual change in enamel | | | |  |  |  | 98 | = extracted for other reason than caries | | | |
| 2 | = sealant, full |  |  | 2 | = distinct visual change in enamel | | | | |  |  | 99 | = unerrupted | |  |  |
| 3 | = tooth coloured restoration |  |  | 3 | = localyzed breakdown in enamel | | | | |  |  | Р = implant or pontic | | | |  |
| 4 | = amalgam restoration |  |  | 4 | = non-cavitated surface with underlying dark shadow of dentin | | | | | | | | |  |  |  |
| 5 | = stainless steel crown |  |  | 5 | = distinct caivty with visible dentin | | | | |  |  |  |  |  |  |  |
| 6 | = porcelain or gold or PFM crown, veneer | | | 6 | = extensive distinct caivty with visible dentin | | | | | |  |  |  |  |  |  |
| 7 | = lost or broken restoration |  |  |  |  |  |  |  |  |  |  |  |  |  |  |  |
| 8 | = temporaty restoration |  |  |  |  |  |  |  |  |  |  |  |  |  |  |  |

1.
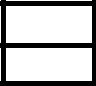
**Score of reported dental fear of the patient by the CFSS-DS**
2. **Treated tooth in this visit (tooth 16 = 1; tooth 26 = 2)**

**8. Pulse frequency** time pulse


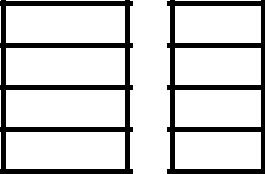


- 1. - mean value in waiting room
  2. - mean value during laser/placebo analgesic procedure
  3. - mean value during treatment
  4. - mean value after treatment before leaving the dental chair

1. **Electric pulp tester values (EPT scores)**
   1. - EPT score 5(five) minutes before laser/placebo analgesic procedure
   2. - EPT score 5(five) minuter after laser/placebo analgesia
   3. - EPT score 20 (twenty) minutes after laser/placebo analgesia
2. **Patient reported score of pain rating sensation after Cold-test on a combination of VAS, NRS and**

**Wong-Baker FACES scaleт:**

- 1. - score 4 (four) minutes before laser/placebo analgesia
  2. - score 6 (six) minutes after laser/placebo analgesia
  3. - score 21 (twentyone) minutes after laser/placebo analgesia

1. **Patient questionaire about the experience of laser/placebo analgesic procedure:**
   1. - yes
   2. - no


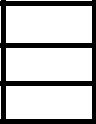

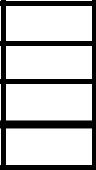

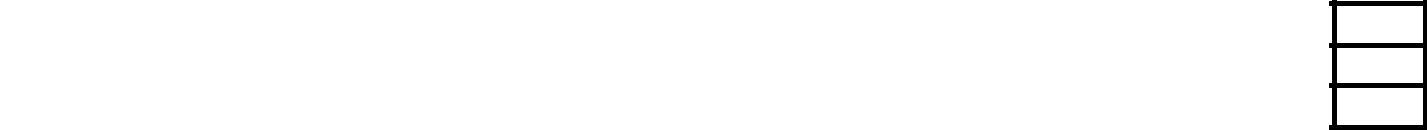


11.1 Did you feel pain while we put your tooth to sleep?

11.2 Were you scared while we were putting your tooth to sleep?

11.3 Do you feel pain now that we have your tooth put to sleep?

11.4 Do you feel any numbness after we have your tooth put to sleep?


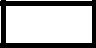


**12. Need for additional anesthesia (local infiltration of anesthetic) during treatment?**

1 - no

2 - yes


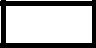


1. **Score of pain felt during treatment of the patient by the VAS, NRS and Wong-Baker Faces Pain Rating Scale, when treatment is finished without need of additional local anesthesia**

**
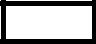
**

**14. Scores of FLACC scale during treatment** total


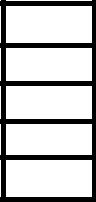


14.1. Score value (face)

14.2. Score value (legs)

14.3. Score value (activity)

14.4. Score value (cry)

14.5. Score value (consolability)

|  |  | | | | | | | | | | | | | | | | | |
| --- | --- | --- | --- | --- | --- | --- | --- | --- | --- | --- | --- | --- | --- | --- | --- | --- | --- | --- |
| **IDENTIFICATION NUMBER** | | | | | | day | |  | month year | | | | | | | operator: |  |  |
|  |  |  |  |  |  |  |  |  |  |  |  |  |  |  |  | outcomes assessor: |  |  |
| **GENERAL INFORMATION** | | | | | |  |  |  |  |  |  |  |  |  |  |  |  |  |
|  | Patient: | | | | |  |  |  |  |  |  |  |  |  |  |  |  |  |
|  | Name: | | | | |  |  |  |  |  |  |  |  |  |  |  |  |  |
| **VISIT** |  |  |  |  | **№** |  |  |  |  |  |  |  |  |  |  |  | 0 | 2 |


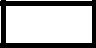


1. **Score of reported dental fear of the patient by the CFSS-DS**
2. **Treated tooth in this visit (tooth 16 = 1; tooth 26 = 2)**

**
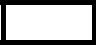
**

**3. Pulse frequency** time pulse


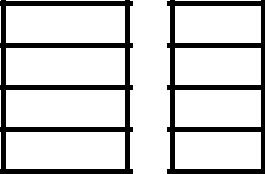


- 1. - mean value in waiting room
  2. - mean value during laser/placebo analgesic procedure
  3. - mean value during treatment
  4. - mean value after treatment before leaving the dental chair

1. **Electric pulp tester values (EPT scores)**
   1. - EPT score 5(five) minutes before laser/placebo analgesic procedure
   2. - EPT score 5(five) minuter after laser/placebo analgesia
   3. - EPT score 20 (twenty) minutes after laser/placebo analgesia
2. **Patient reported score of pain rating sensation after Cold-test on a combination of VAS, NRS and**

**Wong-Baker FACES scaleт:**

- 1. - score 4 (four) minutes before laser/placebo analgesia
  2. - score 6 (six) minutes after laser/placebo analgesia
  3. - score 21 (twentyone) minutes after laser/placebo analgesia

1. **Patient questionaire about the experience of laser/placebo analgesic procedure:**
   1. - yes
   2. - no


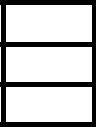

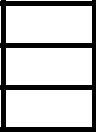

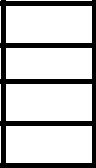


6.1. Did you feel pain while we put your tooth to sleep?

6.2. Were you scared while we were putting your tooth to sleep?

6.3. Do you feel pain now that we have your tooth put to sleep?

6.4. Do you feel any numbness after we have your tooth put to sleep?


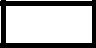


1. **Need for additional anesthesia (local infiltration of anesthetic) during treatment?** (1= no; 2 = yes)
2. **Score of pain felt during treatment of the patient by the VAS, NRS and Wong-Baker Faces Pain Rating Scale, when treatment is finished without need of additional local anesthesia**

**
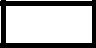

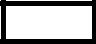
**

**9. Scores of FLACC scale during treatment** total


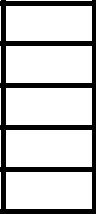


9.1. Score value (face)

9.2. Score value (legs)

9.3. Score value (activity)

9.4. Score value (cry)

9.5. Score value (consolability)
